# Prevalence of the Minimally Conscious State Among Institutionalized Patients in the Netherlands: A Nationwide Study

**DOI:** 10.1212/WNL.0000000000207820

**Published:** 2023-11-14

**Authors:** Berno U.H. Overbeek, Willemijn S. van Erp, Henk J. Eilander, Raymond T.C.M. Koopmans, Jan C.M. Lavrijsen

**Affiliations:** From the Department of Primary and Community Care (B.U.H.O., W.S.v.E., H.J.E., R.T.C.M.K., J.C.M.L.), Radboud University Medical Center, Research Institute of Medical Innovation; Kalorama (B.U.H.O.), Beek-Ubbergen; Azora (B.U.H.O.), Terborg; Accolade Zorg (W.S.v.E.), Bosch en Duin; Libra Rehabilitation & Audiology (W.S.v.E.), Tilburg; and Joachim and Anna, Center for Specialized Geriatric Care (R.T.C.M.K.), Nijmegen, the Netherlands.

## Abstract

**Background and Objectives:**

The minimally conscious state (MCS) is a prolonged disorder of consciousness (pDoC) and one of the most severe outcomes of acquired brain injury. Prevalence data are scarce. The aim of this study was to establish the nationwide point prevalence of institutionalized patients in MCS in the Netherlands.

**Methods:**

This was a descriptive cross-sectional study in which all 86 Dutch hospitals, all 5 specialized pDoC rehabilitation facilities, and all 274 nursing homes were asked whether they were treating patients with a pDoC on the point prevalence date of September 15, 2021. Each patient's legal representative provided informed consent for their inclusion. Patient level of consciousness was verified using the Coma Recovery Scale-Revised (CRS-R) in a single assessment session performed in the facility of residence by an experienced physician. Data on patient demographics, etiology, level of consciousness, facility of residence, and clinical status were collected from a questionnaire by the treating physician. The prevalence of institutionalized patients in MCS of per 100,000 members of the Dutch population was calculated, based on actual census data.

**Results:**

Seventy patients were reported to have a pDoC, of whom 6 were excluded. The level of consciousness was verified for 49 patients while for 15, it could not be verified. Of the patients verified, 38 had a pDoC, of whom 32 were in MCS (mean age 44.8 years, 68.8% male). The prevalence of institutionalized patients in MCS is 0.2–0.3 per 100,000 Dutch inhabitants. Traumatic brain injury was present in 21 of 32 patients (65.6%). Specialized pDoC rehabilitation was received by 17 of 32 patients (53%), with the rest admitted to nursing homes. The most frequent signs of consciousness on the CRS-R were visual pursuit, reproducible movement to command, and automatic motor response.

**Discussion:**

This nationwide study revealed a low prevalence of institutionalized patients in MCS in the Netherlands. These findings are now being used to organize pDoC care in this country.

## Introduction

The minimally conscious state (MCS), in which patients demonstrate inconsistent but observable behavior indicative of consciousness,^1^ is one of the most serious outcomes of acquired brain injury. Together with the unresponsive wakefulness syndrome/vegetative state (UWS/VS), the MCS is part of the spectrum of prolonged disorders of consciousness (pDoCs). Despite the severity of this condition, the epidemiology is largely unknown, hindering the organization of appropriate care.

Data on the prevalence of MCS are scarce. In 2014, 2 systematic reviews about pDoC epidemiology^2,3^ found only 2 studies reporting prevalence data.^4,5^ A 2009 regional French cross-sectional study found a MCS prevalence of 1.9 per 100,000 citizens, with patient's wakefulness evaluated using the Wessex Head Injury Matrix.^6^ A 2011 Austrian study found a MCS prevalence of 1.5 per 100,000 citizens, with a telephone inquiry conducted to determine the level of consciousness (LoC).^5^ On-site LoC verification was not performed.

Diagnosing the LoC is challenging, as reflected by misdiagnosis rates of around 40%.^7,8^ Nevertheless, diagnosing MCS is of great clinical relevance because patients in MCS may have nearly intact pain perception capacity,^9^ which has consequences for pain management. Patients in MCS also have a better prognosis for recovery of consciousness than those in UWS/VS,^10^ and they may benefit more from intensive neurorehabilitation^11^ and other therapeutic interventions.^12^ Finally, medical ethical considerations for these patients can differ from those for patients in UWS/VS.^13,14^

The Netherlands offers optimal possibilities for a nationwide prevalence study into MCS with on-site LoC verification. The Netherlands is a small, densely populated country with approximately 18 million inhabitants.^15^ Hospital and long-term care are well-distributed across the country and easily accessible. From 2019, virtually all new patients with a pDoC are admitted to a chain of care, embedded within a nationwide network of academic expertise. After hospital discharge, early intensive neurorehabilitation (EINP) is offered for up to 14 weeks in a specialized rehabilitation center.^16,17^ EINP patients who do not recover consciousness can continue their rehabilitation in a prolonged intensive neurorehabilitation (PIN) program for up to 24 months after the event, in one of 4 specialized nursing homes.^18^

In this context, we conducted a nationwide point prevalence study on institutionalized patients in MCS.

## Methods

### Study Design

A descriptive cross-sectional study design was chosen because it is the most appropriate method to determine patient prevalence.^19^ In addition, demographic and medical characteristics were recorded. The prevalence was calculated by dividing the number of institutionalized patients by the number of Dutch citizens on the point prevalence date. Actual census data were derived from the population count of the central statistical office in the Netherlands.^15^

### Definitions Used for pDoC, UWS/VS, MCS, and Exit-MCS

A pDoC was defined according to the internationally accepted definition of no or partial return of consciousness ≥28 days after sustaining a brain injury.^20,21^ To identify UWS/VS, the internationally accepted definition and diagnostic criteria reported by the Multi-Society Task Force on Vegetative State were used.^22^ For MCS and exit-MCS, the definitions and diagnostic criteria reported by Giacino et al.^1^ were used. The definitions used are presented in Table 1.

**Table 1:**
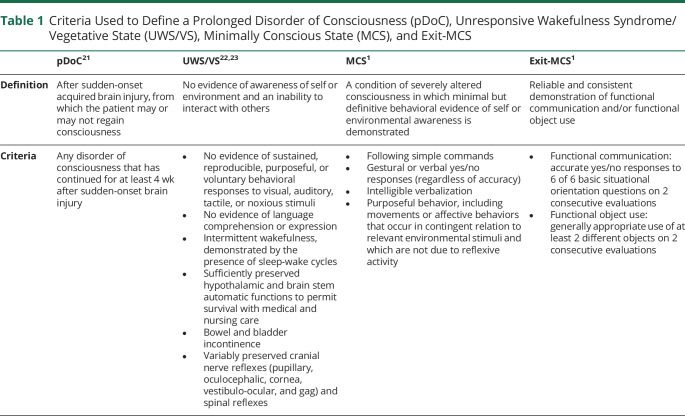
Criteria Used to Define a Prolonged Disorder of Consciousness (pDoC), Unresponsive Wakefulness Syndrome/Vegetative State (UWS/VS), Minimally Conscious State (MCS), and Exit-MCS

	pDoC^21^	UWS/VS^22,23^	MCS^1^	Exit-MCS^1^
Definition	After sudden-onset acquired brain injury, from which the patient may or may not regain consciousness	No evidence of awareness of self or environment and an inability to interact with others	A condition of severely altered consciousness in which minimal but definitive behavioral evidence of self or environmental awareness is demonstrated	Reliable and consistent demonstration of functional communication and/or functional object use
Criteria	Any disorder of consciousness that has continued for at least 4 wk after sudden-onset brain injury	No evidence of sustained, reproducible, purposeful, or voluntary behavioral responses to visual, auditory, tactile, or noxious stimuli No evidence of language comprehension or expression Intermittent wakefulness, demonstrated by the presence of sleep-wake cycles Sufficiently preserved hypothalamic and brain stem automatic functions to permit survival with medical and nursing care Bowel and bladder incontinence Variably preserved cranial nerve reflexes (pupillary, oculocephalic, cornea, vestibulo-ocular, and gag) and spinal reflexes	Following simple commands Gestural or verbal yes/no responses (regardless of accuracy) Intelligible verbalization Purposeful behavior, including movements or affective behaviors that occur in contingent relation to relevant environmental stimuli and which are not due to reflexive activity	Functional communication: accurate yes/no responses to 6 of 6 basic situational orientation questions on 2 consecutive evaluations Functional object use: generally appropriate use of at least 2 different objects on 2 consecutive evaluations

### Identification of Patients

**Figure 1:**
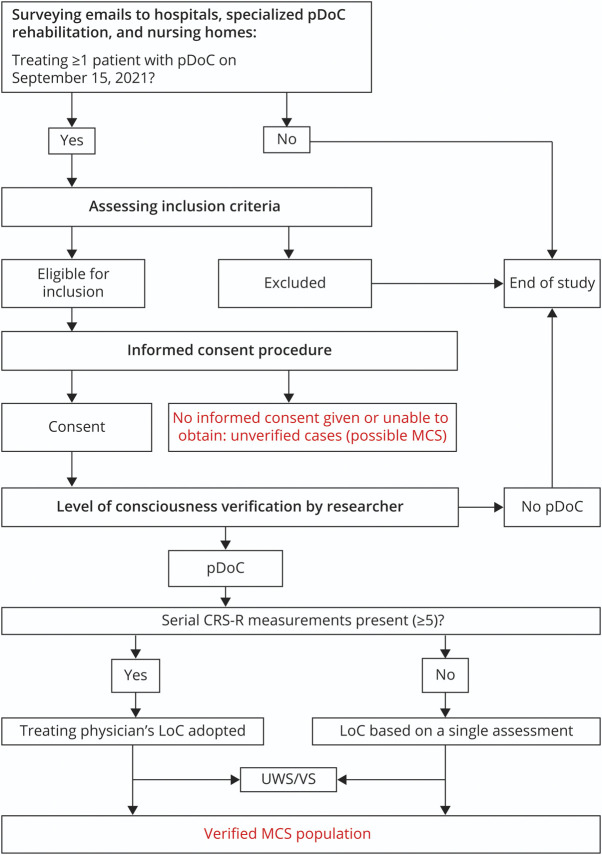
Study Design

The medical directors of all Dutch hospitals with a neurology, neurosurgery, and/or intensive care unit; all institutions that provide specialized pDoC rehabilitation; and all Dutch nursing homes were asked by email whether they had provided care to one or more patients with a pDoC in their department on September 15, 2021, the point prevalence date (Figure 1). We explicitly asked them to report all patients with pDoCs because misdiagnosis of these conditions is very common.^7,8^ The email contained information about the rationale and aim of the study and a link to a fact sheet that explained pDoC and its clinical entities UWS/VS and MCS (eMethods, links.lww.com/WNL/D125). Replies were sent by email. Institutions that did not reply were contacted by telephone. If a response could not be retrieved in this way, the institution/department was registered as a nonresponder.

Inclusion criteria for participation were pDoC after acute acquired brain injury with a duration of ≥4 weeks and a patient aged 16 years or older. Patients with a pDoC due to neurodegenerative disease or malignancy were excluded. If a patient met the inclusion criteria, their representative was asked for written informed consent for LoC verification on site and for the collection of clinical data (Figure 1).

### Data Collection

#### Patient Characteristics

Treating physicians were asked to complete an online questionnaire about the demographic and clinical status of the patients with a pDoC using Castor Electronic Data Capture (EDC).^24^ Concerning LoC, we asked treating physicians to describe the best reaction reported and their LoC diagnosis and to indicate whether this diagnosis was based on random clinical observations, structured scales such as the Coma Recovery Scale-Revised (CRS-R)^25^ or Post-Acute Level of Consciousness Scale-Revised (PALOC-sr),^26^ or other methods.

#### Assessment of the LoC

We aimed to verify LoC on site for all cases in a single assessment using the CRS-R, which can discriminate well between UWS/VS and MCS^25^ and is the most commonly recommended scale for use in daily clinical practice.^27^ LoC verification was performed by the researcher (B.U.H.O., an experienced pDoC clinician). In the assessment, family members and staff were actively involved by providing personally salient stimuli and sharing their own observations of behavior possibly indicative of consciousness, such as context-related emotions and vocalizing or gesturing in response to the linguistic content of a question.^28^ If reported, these were evaluated for contingency in a structured manner as suggested in the CRS-R.^28,29^

We documented the presence of proxies (i.e., representatives of the patients, not necessarily family members) and their relationship to the patient, the patient's posture (i.e., sitting or lying down), and whether the patient had had at least 30 minutes of rest before the assessment. In addition, behavioral reactions were described, and it was noted by whom these reactions were observed. Moreover, the proxies were asked whether the observed reactions were representative of the patient's general functioning. The following possible interfering factors were documented: intercurrent illness, paroxysmal sympathetic hyperactivity, epilepsy, tracheostomy, urinary catheters, use of medication with sedative side effects,^30,31^ and use of artificial nutrition and hydration during the assessment.

#### Case Identification

Based on the findings on the CRS-R, the researcher classified the LoC for each patient as pDoC (i.e., UWS/VS or MCS) or no pDoC (i.e., exit-MCS). Furthermore, MCS was subcategorized into MCS− and MCS+.^32,33^ If the patient had already a pDoC diagnosis based on serial CRS-R assessments (≥5), this diagnosis was adopted. If no serial CRS-R assessment was available, the diagnosis of pDoC was based on the single assessment by the researcher. The rationale for this decision was that serial CRS-R assessments are associated with fewer misdiagnoses.^34^ The total MCS population consists of the verified population plus the unverified cases, who could possibly be in MCS (Figure 1).

### Statistics

The prevalence of institutionalized patients in MCS was expressed as an absolute number per 100,000 Dutch citizens, based on actual census data. Descriptive statistics, including absolute numbers, percentages, means and medians, and interquartile ranges (IQRs), were calculated where applicable using SPSS version 25.0 (IBM, Armonk, NY).

### Standard Protocol Approvals, Registrations, and Patient Consents

The research protocol was reviewed by an accredited medical ethical committee at the Radboud University Medical Center (file number 2020-6169) and was not considered to be subject to the Dutch Medical Research Involving Human Subjects Act (1998). According to the committee, further medical ethical evaluation was not required. Written informed consent was obtained from the legal representatives of all participating patients in the study.

### Data Availability

Anonymized data will be shared on reasonable request.

## Results

### Identification and Verification of Cases

**Figure 2:**
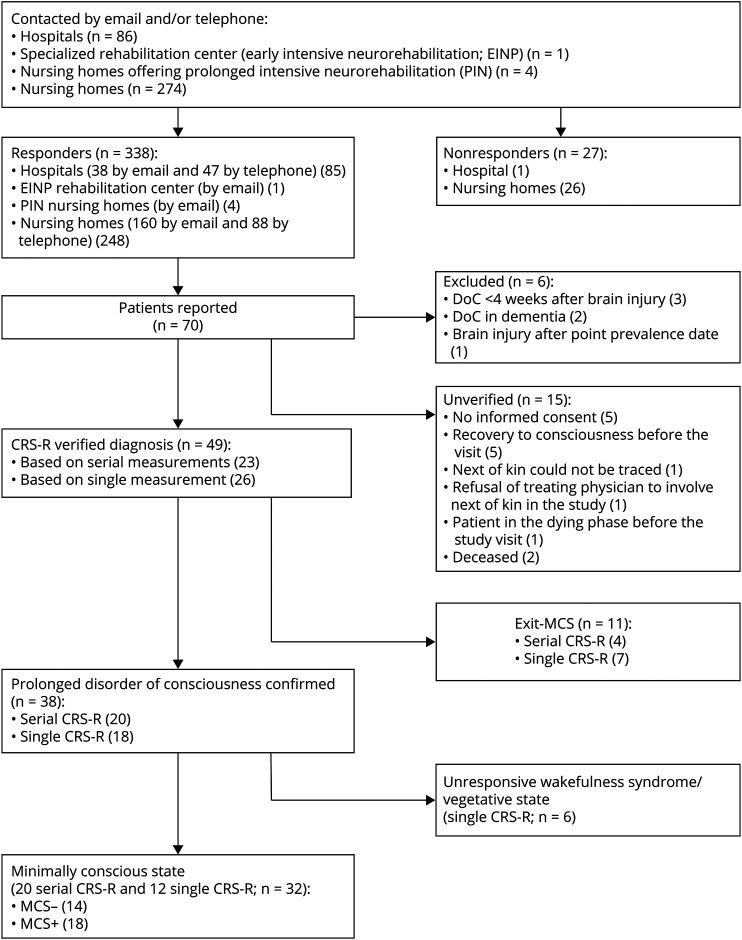
Flowchart of Prevalence Inquiry and Verification

Of the 86 hospitals contacted, 85 (99%) sent at least one response from intensivists, neurologists, or neurosurgeons (Figure 2). All 5 pDoC rehabilitation facilities (EINP and PIN) responded, and of the 274 nursing homes, 248 responded (91%). After informed consent was provided, the patients were visited for LoC verification within a median time lapse of 41 (IQR 14.5–82.0) days.

Treating physicians reported 70 patients as having a pDoC, of whom 64 met the inclusion criteria. The legal representatives of 49 patients gave consent to participate. The LoC of 15 patients, possibly in MCS, could not be verified because of a lack of consent, recovery of consciousness, clinical deterioration, or death before verification. Through verification, 38 of 49 patients (78%) were found to have a pDoC while 11 were in exit-MCS (22%). Of the patients with a pDoC, 6 patients (16%) were in UWS/VS while the majority were in MCS (32; 84%), with 14 in MCS− and 18 in MCS+ (Figure 2).

### Prevalence

The 32 verified and 15 unverified cases correspond to a prevalence of 0.2–0.3 patients in MCS per 100,000 inhabitants of the Dutch general population.^35^

### Signs of Consciousness

The observed signs of consciousness on the single CRS-R assessment were visual pursuit (n = 15 of the patients in MCS), reproducible movement to command (n = 13), automatic motor response (n = 10), object localization (n = 4), intentional communication (n = 3), object manipulation (n = 2), intelligible verbalization (n = 2), and sustained visual fixation (n = 1). Additional findings yielded the following responses: context-related emotions (n = 7; for one patient, 2 different context-related emotions were observed), visual pursuit of TV images and localizing TV sounds (n = 1), finishing a song (n = 1), and anticipation of a moving ball (n = 1) (Table 2). Seventeen patients (53%) showed signs of consciousness on ≥2 subscales; 7 patients (22%) showed only one sign of consciousness, mostly visual pursuit; and 8 patients (25%) showed no signs of consciousness on the single CRS-R assessment. MCS was diagnosed in them based on serial CRS-R assessments or presence of additional behavioral responses indicative of MCS.

**Table 2:**
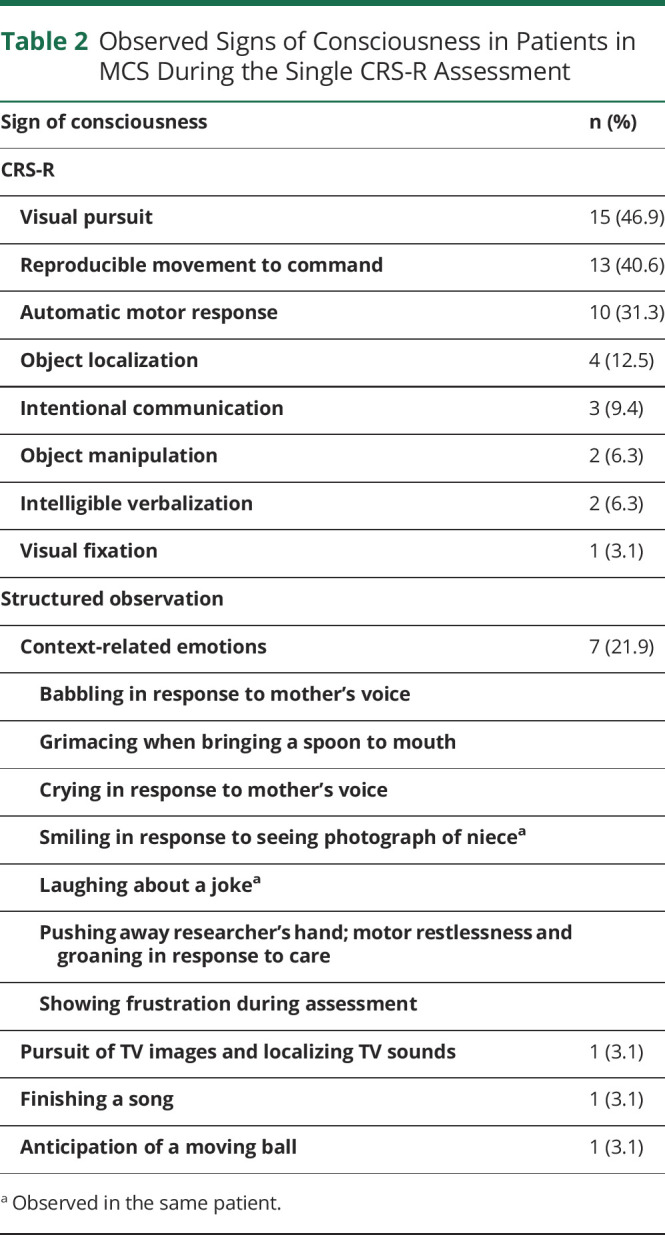
Observed Signs of Consciousness in Patients in MCS During the Single CRS-R Assessment

Sign of consciousness	n (%)
CRS-R	
Visual pursuit	15 (46.9)
Reproducible movement to command	13 (40.6)
Automatic motor response	10 (31.3)
Object localization	4 (12.5)
Intentional communication	3 (9.4)
Object manipulation	2 (6.3)
Intelligible verbalization	2 (6.3)
Visual fixation	1 (3.1)
Structured observation	
Context-related emotions	7 (21.9)
Babbling in response to mother's voice	
Grimacing when bringing a spoon to mouth	
Crying in response to mother's voice	
Smiling in response to seeing photograph of niece^a^	
Laughing about a joke^a^	
Pushing away researcher's hand; motor restlessness and groaning in response to care	
Showing frustration during assessment	
Pursuit of TV images and localizing TV sounds	1 (3.1)
Finishing a song	1 (3.1)
Anticipation of a moving ball	1 (3.1)

a Observed in the same patient.

Differences in LoC diagnoses occurred in 8 patients in MCS (25%) (for more detailed information about the patients, see eTable 1, links.lww.com/WNL/D126). These 8 patients were presumed by their treating physician to be in MCS, but scored UWS/VS on the single CRS-R assessment performed by the researcher. For 7 of them, serial CRS-R assessment scores were available, indicating MCS (6 were in MCS−, one was in MCS+). For one patient in MCS−, serial CRS-R assessments were not performed and MCS− was diagnosed because of the presence of consistent emotional reactions in response to care. Interfering factors were frequently observed in the entire MCS population; in 25 of the 32 patients, one or more possible interfering factors were observed. All 8 patients who scored UWS/VS on the single CRS-R assessment displayed low arousal, indicated by a score of 1 on the wakefulness scale of the CRS-R. Of the other 24 patients in MCS, 20.8% had a low arousal. Other possible interfering factors are shown in the supplemental information (eTable 2, links.lww.com/WNL/D127).

#### Patient Characteristics

The mean age of the institutionalized patients in MCS was 44.8 years; 22 of 32 patients (68.8%) were male, 13 of 32 patients (40.6%) were married, and 5 of 32 patients (15.6%) had a partner (Table 3). The median time elapsed between brain injury and the point prevalence date was 16.5 months (IQR 5.25–52.5), ranging from one month to 17 years. The brain injury underlying the MCS had a traumatic cause in 21 of 32 patients (65.6%), mainly road-traffic accidents or falls. The other cases (11/32; 34.4%) were nontraumatic, mainly caused by postanoxic encephalopathy after out-of-hospital cardiac arrest or subarachnoid hemorrhages. Seventeen patients (53.1%) were admitted to a pDoC rehabilitation facility.

**Table 3:**
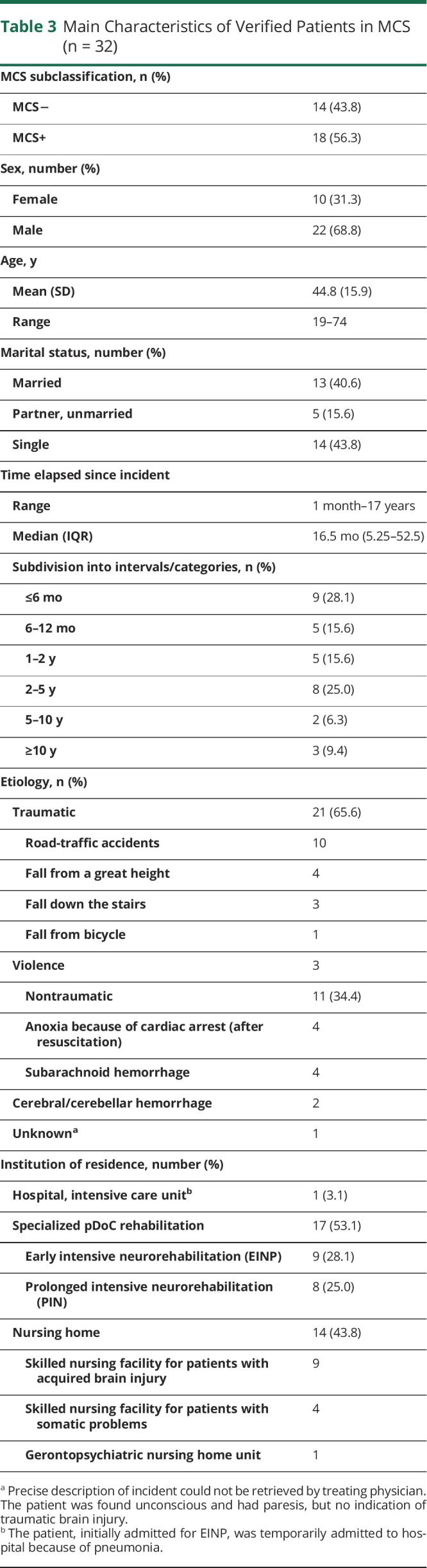
Main Characteristics of Verified Patients in MCS (n = 32)

MCS subclassification, n (%)	
MCS−	14 (43.8)
MCS+	18 (56.3)
Sex, number (%)	
Female	10 (31.3)
Male	22 (68.8)
Age, y	
Mean (SD)	44.8 (15.9)
Range	19–74
Marital status, number (%)	
Married	13 (40.6)
Partner, unmarried	5 (15.6)
Single	14 (43.8)
Time elapsed since incident	
Range	1 month–17 years
Median (IQR)	16.5 mo (5.25–52.5)
Subdivision into intervals/categories, n (%)	
≤6 mo	9 (28.1)
6–12 mo	5 (15.6)
1–2 y	5 (15.6)
2–5 y	8 (25.0)
5–10 y	2 (6.3)
≥10 y	3 (9.4)
Etiology, n (%)	
Traumatic	21 (65.6)
Road-traffic accidents	10
Fall from a great height	4
Fall down the stairs	3
Fall from bicycle	1
Violence	3
Nontraumatic	11 (34.4)
Anoxia because of cardiac arrest (after resuscitation)	4
Subarachnoid hemorrhage	4
Cerebral/cerebellar hemorrhage	2
Unknown^a^	1
Institution of residence, number (%)	
Hospital, intensive care unit^b^	1 (3.1)
Specialized pDoC rehabilitation	17 (53.1)
Early intensive neurorehabilitation (EINP)	9 (28.1)
Prolonged intensive neurorehabilitation (PIN)	8 (25.0)
Nursing home	14 (43.8)
Skilled nursing facility for patients with acquired brain injury	9
Skilled nursing facility for patients with somatic problems	4
Gerontopsychiatric nursing home unit	1

a Precise description of incident could not be retrieved by treating physician. The patient was found unconscious and had paresis, but no indication of traumatic brain injury.

b The patient, initially admitted for EINP, was temporarily admitted to hospital because of pneumonia.

## Discussion

This is a nationwide CRS-R–based prevalence study of patients in MCS in hospitals, specialized pDoC rehabilitation institutions, and nursing homes. We found a prevalence of 0.2–0.3 institutionalized patients in MCS per 100,000 members of the Dutch general population. Most of the MCS population was male, with traumatic brain injury being the most frequent cause. Just over half of the patients were admitted to specialized pDoC rehabilitation institutions.

The prevalence of MCS reported here is 7–10 times lower than that in other countries. In the French Maine-et-Loire region, the MCS prevalence was 1.9 per 100,000 citizens^4^ while in Austria, it was 1.5 per 100,000 citizens^5^; however, these figures cannot easily be compared because of differences in study design. This study investigated the prevalence in a broader population than the other studies; therefore, a higher prevalence would be expected. The lower prevalence could be explained by a more selective enrollment of patients because patients with a pDoC receive specialized care that includes the possibility of diagnosis based on serial assessments. This may have led to a more stringent case ascertainment, which could have influenced the prevalence. In addition, our verification procedure revealed that a substantial number of patients (11/49; 22%) who were reported as being in MCS were, in fact, in exit-MCS. Another explanation could be the difference in end-of-life decisions taken in the acute phase between the Netherlands, France, and Austria. Withdrawal of treatment is more common in intensive care units in northern Europe than in central Europe.^36^ It has been hypothesized before that, in the Netherlands, there is a tendency to discontinue life-sustaining treatment relatively early for patients with severe brain injury. A retrospective Dutch study found that, in patients with traumatic brain injuries who were admitted to an intensive care unit, 82% of those who died did so following a decision to withdraw life-sustaining treatment.^37^ In the postacute and long-term phase, it has been shown that physicians may decide to discontinue life-sustaining therapies for patients with a pDoC, in consensus with the patient's representative.^38^ To determine whether differences in clinical decision making truly account for the variation between the prevalence of pDoC in different countries, international comparative research combining quantitative and qualitative measures would need to be conducted.

This study highlights the importance of conducting serial CRS-R assessments because establishing a diagnosis of MCS was complex in a significant proportion of the identified patient population. A closer look at the entire pDoC population in this study reveals that diagnosing MCS is complex at both the lower (i.e., UWS/VS vs MCS) and upper (i.e., MCS vs exit-MCS) ends of the spectrum of consciousness. In 25% of the population, no signs of consciousness were observed on the single CRS-R assessments, but repeated assessments detected signs of MCS or context-related emotions in structured assessments for contingent behavior. An evaluation of the test conditions revealed that one or more interfering factors were present, mostly a low arousal. This may have contributed to the underestimation of the LoC in these patients because low arousal is a factor known to confound the accurate assessment of the LoC. On the upper spectrum, exit-MCS was not detected in 22% of patients. Diagnostic complexity has also been addressed in recent studies discussing the association of several behavioral signs with a specific LoC, such as the use of auditory localization,^39^ visual pursuit, and visual fixation,^40,41^ to differentiate between MCS− and UWS. In addition, the status of consistent command following for differentiating between MCS+ from exit-MCS has been discussed recently. Consistent command following re-emerges around the same time as functional object use and functional communication, which supports its addition to the exit-MCS criteria.^42^

Relatively few patients in UWS/VS were identified; only 6 of the patients with a pDoC were in UWS/VS, which is 4 to 5 times lower than the number reported in previous Dutch prevalence studies.^8,43^ This low number of patients in UWS/VS has to be interpreted with caution, however, because this study was announced as a nationwide prevalence study on MCS. Although we asked treating physicians to report all patients with pDoC, not exclusively patients in MCS, the focus on MCS may have resulted in a bias in favor of excluding patients in UWS/VS. Furthermore, the tendency to discontinue care to patients in UWS/VS relatively early might have contributed to their low prevalence.^37^ The diagnosis and monitoring of patients with a pDoC has improved in the Netherlands because of their increased access to intensive neurorehabilitation,^17,18^ which may have led to the detection of more patients in MCS, with a possible shift of patients from a UWS/VS to MCS diagnosis. At the time of the 2003 and 2012 UWS/VS prevalence studies, intensive neurorehabilitation was not reimbursed to patients with a pDoC who were 25 years or older, and they were, therefore, admitted to nursing homes after their hospital admission. In nursing homes, LoC was rarely, if ever, subjected to repeated examination. Nevertheless, in the 2003 and 2012 prevalence studies, patients in MCS were also identified.^8,43^ With the further development of specialized treatment of patients with a pDoC, we expect this shift from UWS/VS to MCS to continue. This will need to be confirmed by monitoring patient flow within our network of expertise.

The strength of this study is that the prevalence of MCS has been determined at the national level with the use of expert-based diagnosis verification using the CRS-R. There are, however, limitations to this study. First, inclusion was restricted to institutionalized patients; thus, patients in MCS who were cared for at home or admitted to other facilities, such as institutions for patients with intellectual disabilities, may have been missed. Second, even in this context with serial CRS-R assessments, we must take into account the possibility of having underestimated the LoC in certain patients, for example, because of the presence of possible interfering factors and/or their diagnosis had to be based on a single assessment.

This study demonstrates that a nationwide prevalence study of patients in MCS can be successfully conducted within a specialized chain of expertise, in which pDoC rehabilitation and chronic care are connected. Previously, a vicious circle was described, stating that unknown epidemiology can lead to suboptimal care and a tendency to discontinue treatment in UWS/VS, resulting in low numbers of patients as a consequence.^38^ This study adds up-to-date epidemiologic data, which are indispensable for the organization of pDoC care.

We recommend creating circumstances to facilitate the repetitive assessment of the LoC to obtain reliable epidemiologic data. Furthermore, we recommend the use of a central patient registry to keep epidemiologic data up-to-date and to facilitate longitudinal research. A central registry is currently under development in the Netherlands, and outcome studies into intensive neurorehabilitation are being conducted, which cover both the quantitative and qualitative aspects.^17,18^ Finally, international comparative research into the variation of pDoC prevalence between countries is recommended.

In conclusion, this nationwide study, conducted in a setting where care, rehabilitation, and research are connected, reveals a low prevalence of institutionalized patients in MCS. This prevalence is being used to further develop and optimize pDoC care in the Netherlands.

## Appendix Authors

**Table:**

Name	Location	Contribution
Berno U.H. Overbeek, MD	Department of Primary and Community Care, Radboud University Medical Center, Research Institute of Medical Innovation; Kalorama, Beek-Ubbergen; Azora, Terborg, the Netherlands	Drafting/revision of the manuscript for content, including medical writing for content; major role in the acquisition of data; study concept or design; analysis or interpretation of data
Willemijn S. van Erp, MD, PhD	Department of Primary and Community Care, Research Institute for Medical Innovation; Accolade Zorg, Bosch en Duin; Libra Rehabilitation & Audiology, Tilburg, the Netherlands	Drafting/revision of the manuscript for content, including medical writing for content; major role in the acquisition of data; study concept or design; analysis or interpretation of data
Henk J. Eilander, PhD	Radboud University Medical Center, Research Institute of Medical Innovation	Drafting/revision of the manuscript for content, including medical writing for content; major role in the acquisition of data; study concept or design; analysis or interpretation of data
Raymond T.C.M. Koopmans, MD, PhD	Radboud University Medical Center, Research Institute of Medical Innovation; Joachim en Anna, center for specialized Geriatric Care, Nijmegen, the Netherlands	Drafting/revision of the manuscript for content, including medical writing for content; major role in the acquisition of data; study concept or design; analysis or interpretation of data
Jan C.M. Lavrijsen, MD, PhD	Radboud University Medical Center, Research Institute of Medical Innovation	Drafting/revision of the manuscript for content, including medical writing for content; major role in the acquisition of data; study concept or design; analysis or interpretation of data

## Glossary

CRS-R: Coma Recovery Scale-Revised
EINP: early intensive neurorehabilitation
IQR: interquartile range
LOC: level of consciousness
MCS: minimally conscious state
pDoC: prolonged disorder of consciousness
PIN: prolonged intensive neurorehabilitation
UWS/VS: unresponsive wakefulness syndrome/vegetative state
